# Supplementary material for: The Strong Cell-based Hydrogen Peroxide Generation Triggered by Cold Atmospheric Plasma
Source: Sci Rep. 2017 Sep 7;7:10831. doi: 10.1038/s41598-017-11480-x (PMC5589829; doi:10.1038/s41598-017-11480-x)
Supplement: Supplementary file 1 — Fig. S1, Fig. S2, Fig. S3. [file 41598_2017_11480_MOESM1_ESM.pdf]

The Strong Cell-based Hydrogen Peroxide Generation Triggered by Cold Atmospheric Plasma

Dayun Yan[1*], Haitao Cui[1], Wei Zhu[1], Annie Talbot[2], Lijie Grace Zhang[1], Jonathan H. Sherman[3], and Michael Keidar[1*]

[1]Department of Mechanical and Aerospace Engineering, The George Washington University, Science & Engineering Hall, 800 22nd Street, NW, Washington, DC 20052, USA

[2]Columbian College of Arts and Sciences, The George Washington University, Philips Hall, 801 22nd Street, NW, Suite 212, Washington, DC 20052, USA

[3]Neurological Surgery, The George Washington University, Foggy Bottom South Pavilion, 22nd Street, NW, 7th Floor, Washington, DC 20037, USA

*Corresponding authors: Dayun Yan ydy2012@gwmail.gwu.edu, Michael Keidar keidar@gwu.edu

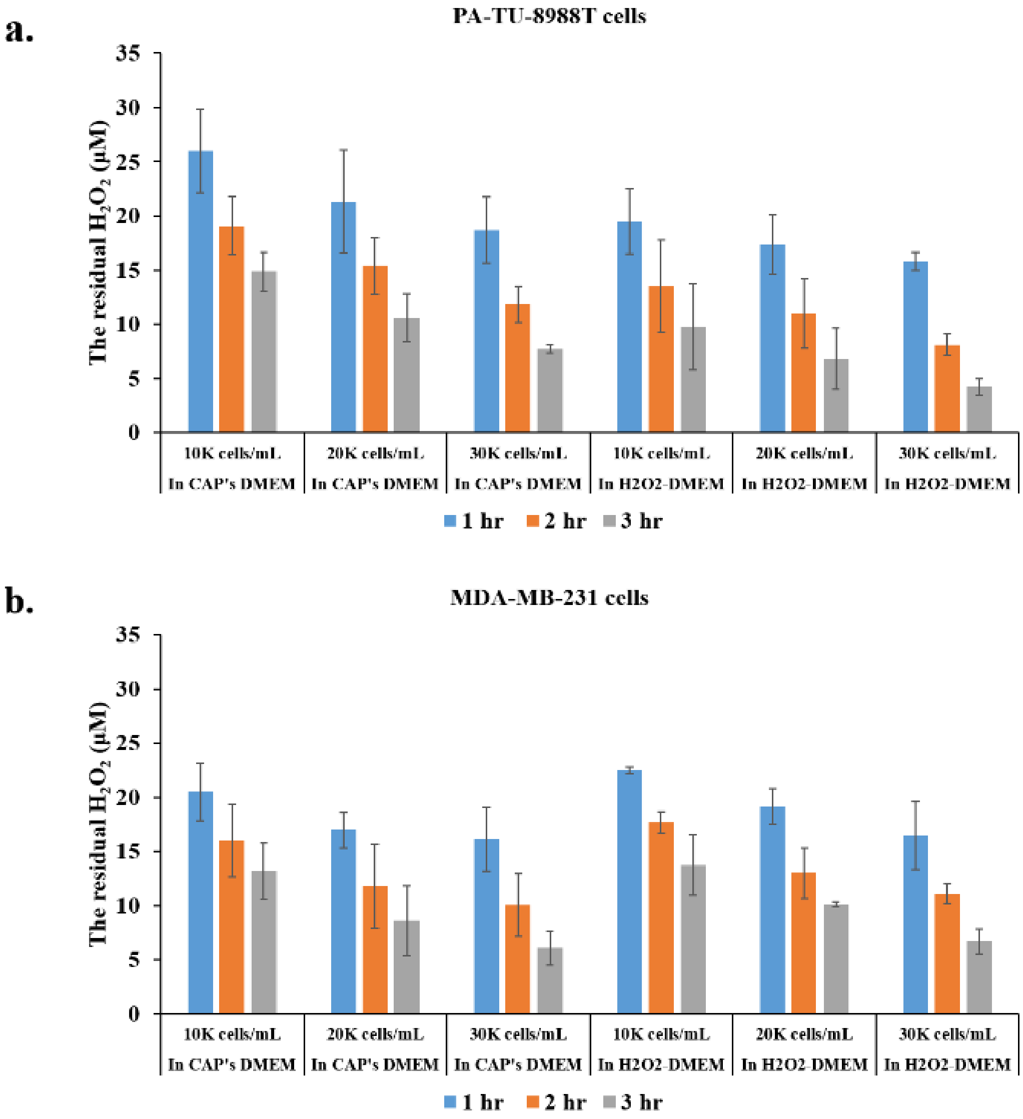

Fig. S1. Cancer cells quickly consume the H_2O_2 in CAP-stimulated DMEM (CAP's medium) and in H_2O_2-containing DMEM (H_2O_2-DMEM) in hours. (a) PA-TU-8988T cells. (b) MDA-MB-231 cells. Consuming H_2O_2 is a basic feature of cancer cells exposed to H_2O_2. 1 hr, 2 hr, and 3 hr represent the time length that two cell lines cultured in CAP's DMEM or in H_2O_2-DMEM. The initial H_2O_2 concentration in CAP's medium and H_2O_2- DMEM is the same and is not shown at

here. K represent 1 x 10^3. Results are presented as the mean ± s.d. of two independently repeated experiments in triplicate.

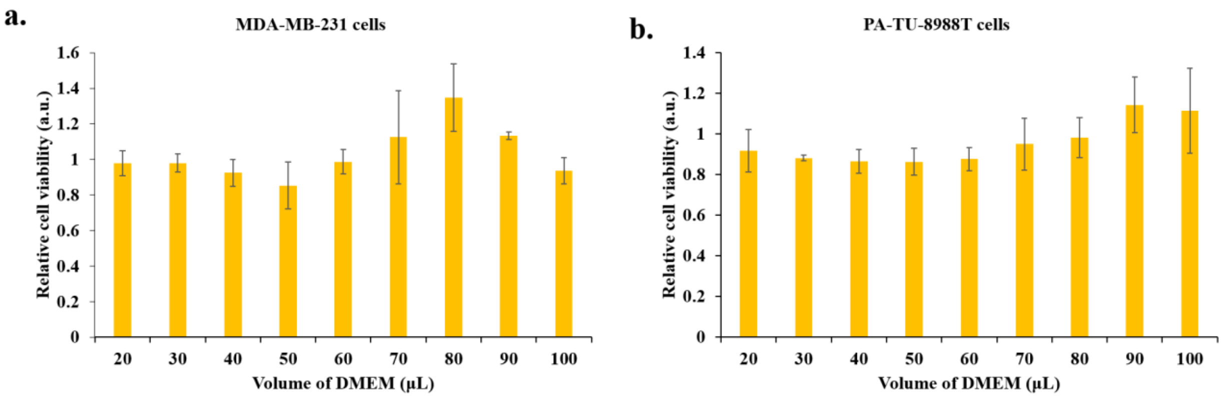

Fig. S2. The effect of helium flow on the cell viability of cancer cells. (a) PA-TU-8988T cells. (b) MDA-MB-231 cells. Results are presented as the mean ± s.d. of three independently repeated experiments.

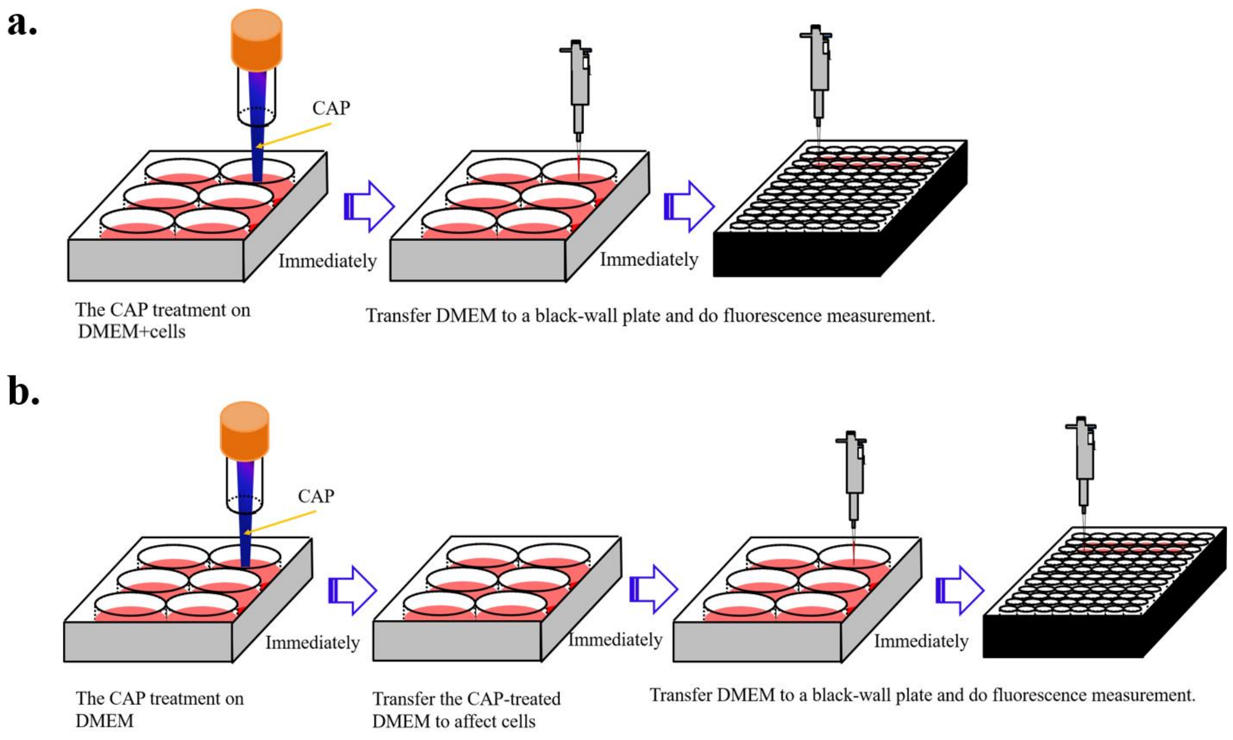

Fig. S3. Schematic illustration for the protocols of CAP treatment. (a) Direct CAP treatment. (b) Indirect CAP treatment.
